# T regulatory cells metabolism: The influence on functional properties and treatment potential

**DOI:** 10.3389/fimmu.2023.1122063

**Published:** 2023-03-03

**Authors:** Martyna Tomaszewicz, Anna Ronowska, Maciej Zieliński, Agnieszka Jankowska-Kulawy, Piotr Trzonkowski

**Affiliations:** ^1^ Department of Medical Immunology, Faculty of Medicine, Medical University of Gdańsk, Gdanísk, Poland; ^2^ Poltreg S.A., Gdanísk, Poland; ^3^ Department of Laboratory Medicine, Faculty of Medicine, Medical University of Gdańsk, Gdanísk, Poland

**Keywords:** metabolism, autoimmunity, glycolysis, fatty acid oxidation, mTOR, ischemia, mitochondria, adenosine

## Abstract

CD4^+^CD25^high^FoxP3^+^ regulatory T cells (Tregs) constitute a small but substantial fraction of lymphocytes in the immune system. Tregs control inflammation associated with infections but also when it is improperly directed against its tissues or cells. The ability of Tregs to suppress (inhibit) the immune system is possible due to direct interactions with other cells but also in a paracrine fashion *via* the secretion of suppressive compounds. Today, attempts are made to use Tregs to treat autoimmune diseases, allergies, and rejection after bone marrow or organ transplantation. There is strong evidence that the metabolic program of Tregs is connected with the phenotype and function of these cells. A modulation towards a particular metabolic stage of Tregs may improve or weaken cells’ stability and function. This may be an essential tool to drive the immune system keeping it activated during infections or suppressed when autoimmunity occurs.

## Graphical Abstract

T regulatory lymphocyte metabolic pattern (1–6). [The red arrow (-) shows inhibition of the process/receptor expression, the green arrow (+) shows stimulation of the process/receptor expression. FoxP3 transcriptional factor is responsible for suppressive function of T regulatory cells. Mitochondrial metabolism include FAO, TCA, and OXPHOS. *The activity of the NF-κB pathway and hypoxia influence glycolysis intensity, GLUT1, and FoxP3 expression through hypoxia inducible factor 1α (HIF-1α)].

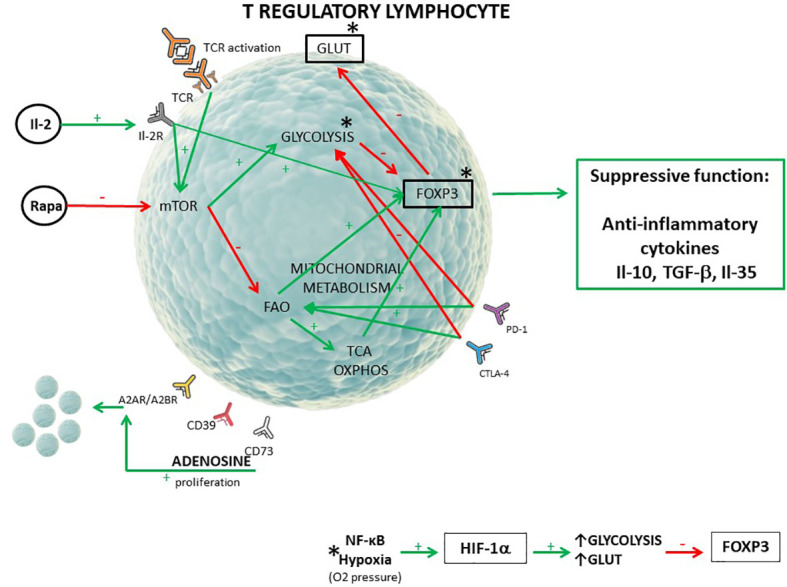

## Introduction

Like any other live cells, immune cells are driven by metabolic machinery. It may be assumed that particular subsets of the immune system differ in activity due to different metabolic pathways utilized during the immune response. There is also growing evidence that genetic mutations can up- or downregulate particular metabolic pathways and adjust metabolism to meet the requirements of the cell (7). This observation initiated a series of studies that aimed to understand the signalling pathways responsible for the controlling metabolic processes. In this review, we try to address whether the regulatory function may be linked to the metabolism of T regulatory cells (Tregs) and if modulation of the cell metabolome can be then utilized to control cell function and, more precisely, to treat human diseases (7).

## Metabolomics- metabolome analysis

In the last few years, metabolomics, defined as the profiling of metabolites in biofluids, cells, and tissues, has become increasingly important in science (8).

Metabolomics may be represented as a concentration of metabolites or, indirectly, enzyme activities, which particularly visualize the processes of cell activity (9). Additionally, cell metabolism is inextricably linked to other parts of the cellular machinery, such as proteomics and genomics. Any changes in the body may result in a series of metabolic shifts. Consequently, metabolic patterns carry an abundance of information that may influence their phenotype and function (10). Through these interactions, metabolites become direct modulators of biological processes (9). This information revealed countless insights into creating active structures that can influence various cellular pathways (9).

Historically, one of the first observations in the field of metabolomics was the Warburg effect, made by Otto Warburg, which states that cancer cells rely on less sufficient aerobic glycolysis despite oxygen availability (11). Although the first suggestion was that the mitochondrial function of cancer cells was defective, further research indicated that there was no switching from mitochondrial respiration to alternative glycolysis in cancer (7). Moreover, oxidative phosphorylation (OXPHOS) continues normally, producing adenosine triphosphate (ATP) like in normal tissues under an identical partial pressure of oxygen (12). On the other hand, this effect plays a vital role in the metabolic reprogramming of cancer cells, which is considered a ‘hallmark of cancer’ (13). Therefore, it is not only a simple adaptation to hypoxia- it is a crucial feature of the malignant phenotype, helping to supply more energy for the overgrowth of tumour cells.

Although the composition of the human metabolome is still not yet fully defined, compelling evidence indicates that the metabolism of immune cells is a critical control point for subsequent migration, proliferation, differentiation, and maintenance of function. A key finding is that a cell phenotype and its metabolic program are correlated with cell function and, importantly, that changing the metabolic state of the cell can cause a change in its functioning (14). The knowledge of how to orchestrate immune cell metabolism may be key to directing the immune system’s potential in treating autoimmune diseases, allergies, or transplant rejection.

## T regulatory cells

As the name suggests, Tregs are a unique population of cells that regulate and suppress other cells in the immune system promoting immune tolerance to self and foreign antigens. We can characterize them as CD4+, CD25^high^, CD127-, and FoxP3+ (Forkhead box P3) cells (15). Tregs express GLUT family member transporters, mainly GLUT1, and GLUT3 (16, 17).

Forkhead box P3 is the most essential and characteristic protein expressed by the Tregs. The level of FoxP3 expression is associated with the maturation process, cell stability, and suppressive function of Tregs (18). Activated T regulatory cells produce suppressive cytokines such as Interleukin 10 (Il-10), Transforming Growth Factor β (TGF-β), and immunomodulatory adenosine (ADO) (18, 19). Treg lymphocytes lacking FoxP3 expression are associated with reactions against self-antigens. For example, a genetic loss-of-function mutation in the gene encoding FoxP3 induces IPEX syndrome (Immune Dysregulation, Polyendocrinopathy, X-linked) manifested as a complex autoimmune syndrome (20).

Metabolic requirements depend on the cell type and on their activity state. Although the metabolic processes of effector T cells are quite well known, understanding mechanisms in Tregs have not been fully elucidated. It is not clear how changing energy sources affects the immunophenotype and stability of Tregs.

### Metabolic differences between resting and activated cells

The basic metabolic program depends on the cell’s activity state and varies between naive, activated, and memory cells.

**Figure 1:**
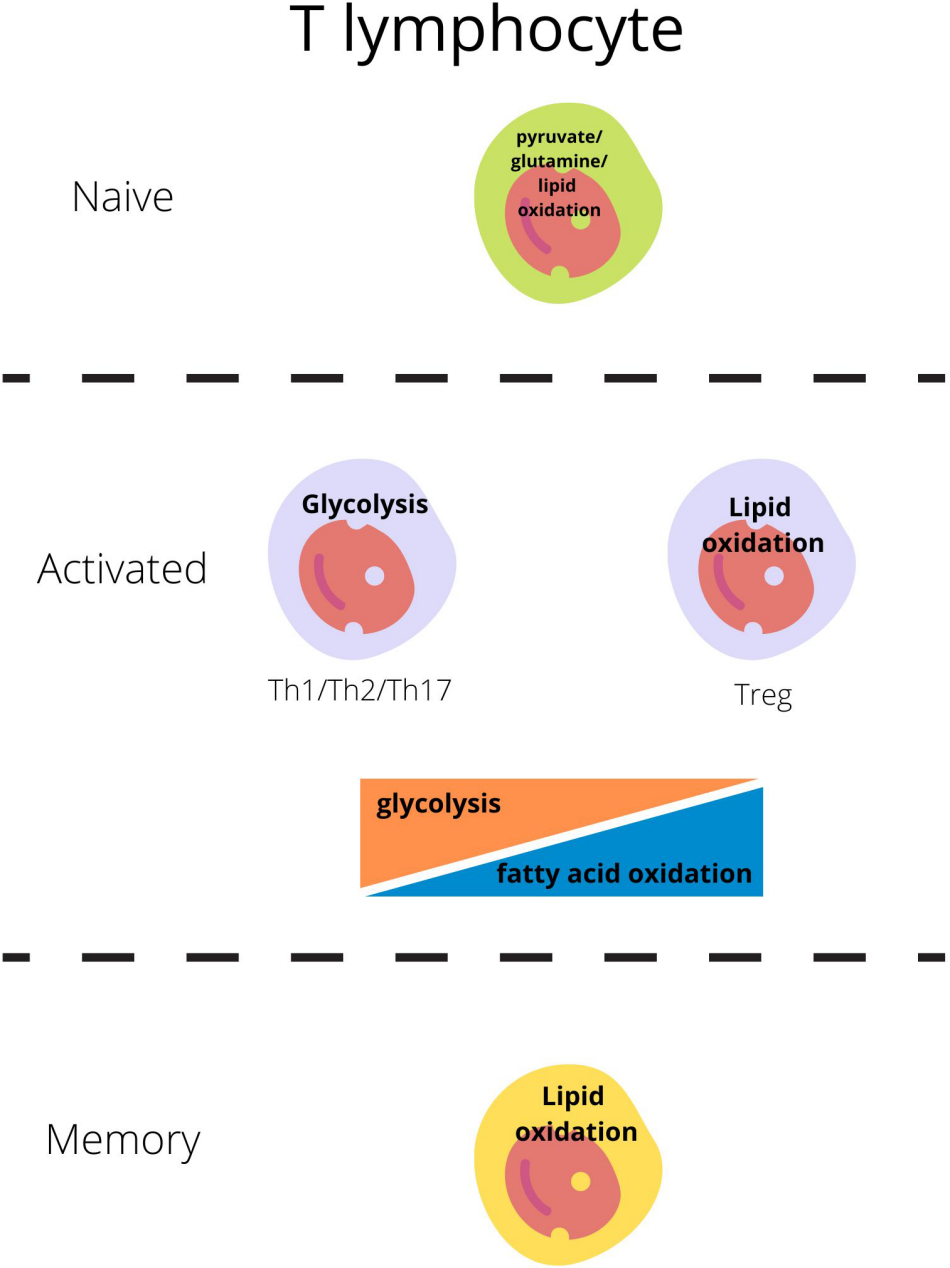
Main metabolic pathways responsible for energy production in T lymphocytes regarding their activity state (21, 22).

Naive-resting cells use the energy sources (ATP) produced mainly through oxidative phosphorylation (OXPHOS) from the lipids or glucose as they need energy only for survival and circulation (**Figure 1**) (23, 24).

Each subset of T lymphocytes generates its specific metabolic pattern. T effector cell (Teff; including Th1, Th2, Th17) activation through the T-cell receptor (TCR) and costimulatory CD28 is the signal for the cell to switch metabolism from OXPHOS to glycolysis which will be sufficient for cell growth, proliferation, and cytokine production (1, 4, 24–26). Teffs, upon activation, remain on glycolysis but also glutaminolysis to generate ATP through intense production in the tricarboxylic acid cycle (TCA) (**Figure 1**) (23, 24).

On the other hand, Tregs engage glycolytic metabolism during initial activation, migration, and proliferation, but subsequently, exhibit oxidative metabolism dependent on lipids and pyruvate [fatty acid oxidation- FAO and oxidative phosphorylation-OXPHOS] and become independent of glucose (**Figure 1**) (27, 28).

Changing energy sources is the process which supports their suppressive activity (23).

Proliferating Treg cells are characterized by elevated GLUT1 expression and mTOR (mammalian target of rapamycin) activity, although their suppressive capacity is then reduced, simultaneously downregulating FoxP3 expression (29). Additionally, Kishore et al. in their research confirmed that stimulation of the pro-migratory molecule Lymphocyte function-associated antigen 1 (LFA-1) with its ligand (antibody-ligated recombinant mouse ICAM-1; rICAM-1) significantly increases glucose uptake to the cell (30).

Fatty acid oxidation in cultured mice Tregs is regulated by AMP-activated kinase (AMPK). In contrast to mTOR kinase, this metabolic regulator influences lipid oxidation and inhibits *de novo* fatty acid synthesis (31).

Within cells, fatty acid binding proteins (FABPs) are responsible for fatty acid uptake and trafficking. In Treg cells, mainly FABP5 is expressed. *In vitro* experiments on Tregs induced from human CD4+ cells with FABP5 inhibition resulted in dysregulation of mitochondrial function, decreased OXPHOS, and as compensation, upregulations of the glycolytic pathway (6). Interestingly, after treatment with FABP5 inhibitor, Tregs had an increased ability to suppress CD4+ and CD8+ effector cell proliferation and were characterized by a higher Il-10 expression (6).

Most quiescent memory immune cells reduce the intensity of metabolic processes. Like the naive cells, these long-lived cells remain in a steady state and use primarily fatty acids to fuel oxidative phosphorylation and generate ATP to cover basic needs (26, 32).

### Differences between central and tissue resident Tregs

Within the Treg population, heterogeneous subpopulations with different phenotypes and functions can be distinguished. After maturation in the thymus, naive-like central cells egress the thymus and inhabit the peripheral lymphatic organs (cTregs) (33). Upon antigen stimulation, circulating in peripheral lymphoid organ cTregs may be differentiated into effector Tregs (eTreg) and migrate to the peripheral tissues (34). As mentioned, cTregs and eTregs may vary in phenotype and function. Moreover, many tissue-specific factors (e.g. oxygen availability, nutrient and metabolites) may change peripheral cells’ characteristics (35). eTregs can expand and suppress more effectively by dynamically regulating specific metabolic pathways, including mTOR (33).

For example, adipose tissue Tregs aim to inhibit proinflammatory processes in fat and are responsible for sustaining balanced insulin sensitivity, whereas Tregs in skeletal muscles or the brain are responsible for amphiregulin (AREG) production and participate in after-injury tissue regeneration (33).

Furthermore, specific bacterial-derived metabolites influence gastrointestinal Tregs.

Smith et al. in their research on mouse models demonstrated that treatment with short-chain fatty acids (SCFA) *in vivo* increased cTregs FoxP3 and Il-10 expression. Additionally, *in vitro* experiments assuming Treg treatment with SCFA resulted in an improvement of Treg proliferative capacity and suppressive activity (36).

As a result, the tissue environment is the primary factor behind metabolism orchestration and Treg diversification in non-lymphoid tissues (33).

## Intrinsic factors affecting cell function

### Glucose-lipid balance and the influence on Treg stability

The activation of human Tregs with toll-like receptor (TLR) agonists or forced expression of GLUT1 increases glycolysis and proliferation but inhibits their anti-inflammatory suppressive functions (18, 28). On the other side, when glucose is deprived in inflammatory conditions, it shifts the balance between Teffs (T effector cells) and Tregs, preventing Th lymphocyte differentiation and reciprocally favouring Tregs development both *in vitro* and *in vivo* (37).

Hyperglycemic conditions induce inflammatory gene expression in lymphocytes, including Il-6, Il-9, and Il-17, which are mostly NF-κB dependent (38).

Moreover, hyperactivation and oxidative stress caused by high glucose levels lead to autoregressive reactions and disruptions in tolerance induction by Tregs. The presented mechanism may be a reason behind propelling of inflammation and exacerbation of the disease in poorly controlled diabetic patients (39).

The mechanism which may secure activated Tregs from elevated glycolysis is the reduction of GLUT1 expression exerted by the transcriptional factor FoxP3 (essential Tregs marker). This subsequently reduces the transport of glucose to the cell and improves the suppressive function of Tregs (18).

On the other hand, Treg differentiation was activated by diacylglycerol O-acyltransferase 1 (DGAT1) - mediated TAG synthesis (40). The decrease in lipid droplets increases the generation of FoxP3+ Tregs in the presence of α-ketoglutarate. Some studies have shown that α-ketoglutarate increases interferon γ (IFN-γ) secretion by CD4 T cells as well as CD4+ CAR-T cells, irrespective of whether they are activated in Treg or Th1 conditions (40) (41).

Additionally, the experiment with etomoxir treatment, which is the blocker of lipid oxidation through inhibition of carnitine palmitoyltransferase-1 (CPT1), transporter of fatty acids into the mitochondria, indicated that this compound decreases the suppression exerted by Tregs differentiation, while not changing T effector lymphocyte functioning (42). This notion proves that elevated glycolysis impairs mainly the activation of Tregs (27, 43, 44).

Cytotoxic T cell antigen 4 (CTLA-4) also conditions the interplay between glycolysis and

T regulatory cell’ function. The blockade of this receptor increases glucose metabolism and promotes Treg functioning instability (45). Research showed that CTLA-4 stimulation does not affect the transport of glucose on its own but may inhibit glucose uptake induced by CD28-stimulated cell activation, making glycolysis cease to be the main energy source in the Treg cell (30).

Finally, our recent studies suggest that Tregs may keep independence from glycolysis and continue metabolism based on OXPHOS through the active uptake of mitochondria from surrounding cells, mainly mesenchymal stem cells (MSCs). These ‘borrowed’ mitochondria are usually active producers of ATP from OXPHOS and improve the function of Tregs (46).

### mTOR

The mammalian target of rapamycin (mTOR) is a protein kinase that belongs to the phosphatidylinositol 3-kinase-related kinases (PIKK) family (47, 48). mTOR controls crucial cell processes such as transcription, translation, cell growth, and metabolism in response to multiple factors (nutrients, growth factors, hormones, and stresses) (49, 50). For this reason, any modulation of the mTOR pathway significantly changes the function of the cells, which is associated with a probability of mutagenesis, autoimmunological reactions, or other types of immune diseases (50, 51). While the activity of mTOR is necessary for Teffs (Th1, Th2, Th17) differentiation, mTOR deficient T cells keep T regulatory cell phenotype instead (1, 52).

The hyperactivation of mTOR kinase in Tregs, through enhancement of glycolytic programming, may result in reduced proliferative capacity (defective function) and promote cell anergy (53, 54).

However, suppression of mTOR kinase signalling in Tregs promotes mitochondrial oxidative metabolism rather than glycolysis, improving suppressive function (42). Hence, excessive mTOR activation can also lead to impairment in Treg survival, and lineage instability manifested mainly through low FoxP3 expression (55, 56). This is widely used in the modulation of Tregs in experimental and clinical settings.

## Extrinsic factors affecting cell function

### Ischemic environment and inflammation

It is not only the life cycle of a cell that causes metabolic shifts. Recent studies have shown that immune cells function differently depending on the environment in which they reside.

Ischemic tissues show low glucose and high lactate concentrations, which seems inappropriate for T lymphocytes relying on oxidative glucose metabolism. Although, it turns out that T regulatory cells are becoming highly pro-tolerant then. The mechanism of this process is still unknown, but it may be associated with Hypoxia Inducible Factor 1 (HIF-1) and its influence on FoxP3 expression (28, 43, 44).

The end-product of anaerobic glycolysis, lactate, in physiological concentration in blood and healthy tissues reaches approximately 1.5–3 mM. Lactate, at a physiologic 1mM concentration, can substitute only about 10% glucose. However, during lactic acidosis evoked by hypoxia, diabetes, chronic obstructive lung disease, disseminated cancer, or inherited metabolic diseases, blood lactate may reach 10 mM and higher concentrations and replace up to 25% glucose in acetyl-CoA and energy production (57). There are data showing that its level may reach 10–40 mM in inflamed tissues such as arthritic joints and adipose tissue in obese individuals (58, 59). Lactate is transported by solute carrier transporters that perform proton-lactate symport, monocarboxylate transporter 4 (MCT4) transporter of low affinity (Km 22 -28 mM) and sodium-dependent transport (SLC5A8) (58). CD4+ T cells do express SLC5A12 transporter (58). The experiments demonstrated that lactate accumulation in the inflamed tissue contributes to the upregulation of the SLC5A12 transporter in human CD4+ T cells (59). Therefore, lactate is a major signalling molecule able to operate the plastic shift of the immune response within the diseased site. Also, lactate may increase inflammation by the elevation of IL-6 and IL-8 secretion by CD4+ T cells (57). Moreover, a lactate receptor,

G protein-coupled receptor 81 (GPR81), was identified on CD4+ T cells (60, 61). Binding of lactate to this receptor led to the activation of PD-L1, which resulted in T effector cell suppression (61).

Nevertheless, lactate is often considered a side product of glycolytic metabolism. Findings show that deficiency of monocarboxylate transporter 1 (MCT1; high affinity lactate and pyruvate transporter) may impact Treg cell function in the gut. Because lactate not only fuels the TCA cycle but is also exported from the mitochondria, contributing to higher glycolytic pathways *via* phosphoenolpyruvate (PEP). It is produced from malate to oxaloacetate and catalyzed by carboxykinase (PEPCK). Thus lactate may become a gluconeogenic fuel source, leading to a decrease in glucose uptake by Treg, resulting in increased Treg cell suppressor function (62).

The hypoxia-inducible transcription factor (HIF-1) is one of the most vital elements in physiological adaptation to varying oxygenation states. It is expressed in almost all mammalian cell types and plays an important functional role in innate and adaptive immune cells, including macrophages, neutrophils and lymphocytes (63–65). HIF transcription factor is controlled by many other factors besides oxygen. For instance, cytokines can induce HIF-1 in response to inflammation and infection of the immune system. In particular, in T cells, HIF-1 is stabilized upon TCR activation, which plays a role in the metabolic transition to glycolysis, which in turn supports proliferation and effector function. There is strong evidence that it regulates glycolysis through the activation of hexokinase and phosphofructokinase but also induces overexpression of GLUTs on the cell surface (66, 67). Another research group carried out an experiment on mice models to check the interplay between FoxP3 and HIF-1α, and the results showed that HIF-1α enhances FoxP3 ubiquitination and degradation (68). Glycolysis, subsequent lactate production, and its accumulation lead to tissue acidosis (69). Interestingly, there are reports that the end-product of glycolysis, lactate, causes decreasing pH in inflamed tissues, inhibits T cell migration, increases proinflammatory Il-17 production, and results in cytolytic activity by CD4+ and CD8+ T cells (25).

Another transcriptional factor mediating inflammatory responses is Nuclear factor kappa-light-chain-enhancer of activated B cells (NF-κB). This protein complex induces the expression of proinflammatory genes (Il-6, Il-18) and participates in inflammasome regulation (70). As a consequence, Teffs are recruited to the inflamed tissues, which allows them to fight off the cause of the infection. However, when the immune response is dysregulated, it contributes to the development of chronic inflammation and tissue damage (70). In addition, it was confirmed that NF-κB induces the expression of HIF-1α (71, 72). The increased levels of NF-κB may lead to the enhancement of glycolytic metabolism and the production of proinflammatory cytokines, chemokines, and angiogenic factors through the induction of the HIF-1*a* pathway linking innate immunity and hypoxic response (72). This mechanism will also adversely affect Tregs, impairing their ability to suppress enhanced proinflammatory reactions.

Summarizing the above mechanisms, it appears that selective glucose deprivation or reduction of the glycolysis-enhancing pathways intensity in Treg cells can contribute to inhibition of inflammation and excessive stimulation of T effector cells.

### Adenosine

An inflammatory immune response is inextricably linked with the release of high amounts of ATP into the extracellular space, where it is captured and partially transformed into adenosine (ADO) (5).

ADO is considered to be a crucial metabolic immune checkpoint that regulates inflammatory responses. Adenosine receptors (ARs; A1R, A2AR, A2BR, and A3R) are expressed in all immune cells (73). The interaction of ADO with its surface receptor triggers anti-inflammatory effects. Studies on Tregs report that these cells express CD39 (ectonucleoside triphosphate diphosphohydrolase-1) and CD73 (ecto-5’-nucleotidase) molecules which are the cell-surface enzymes converting ATP into adenosine through a series of dephosphorylation of the adenine nucleotides (74). Deaglio et al. showed that FoxP3+CD39+ Tregs effectively suppress proliferation of CD4+CD25- effector T cells through the interaction of ADO derived from CD39 and CD73 expressed on Tregs with A2 adenosine receptor on the cell surface (75) and subsequent cAMP production (5, 76). Additionally, FoxP3 is associated with CD39 expression, which also links ADO metabolism with the function of Tregs (5, 77, 78). ADO influences immune responses by altering the release of cytokines. For example, the ligation of ADO to its receptor upregulates the production of Il-10 in experimental mouse models (79). Interestingly, ADO has also been shown to increase the amount of Tregs through ADO-A2AR interaction (80).

Considering the above research, adenosine carries great potential in increasing Treg’s functional potential. Finding a way to more effectively influence ADO-AR pathways may be a possible step toward designing new *in vivo* and *in vitro* treatment methods by influencing Treg-Teff and Treg-Treg interactions (increased production of suppressive cytokines, strengthening the effect of ADO on Teffs, or increased proliferation of Tregs).

## Treatment with Il-2

T cells may be activated in a few different ways, *inter alia* by triggering TCR, TLR, or CD25. CD25 (also known as Il-2Rα, Interleukin-2 Receptor α) is a high affinity molecule present on the T cell surface responsible for interaction with Interleukin 2 (Il-2).

Research suggests that Il-2 promotes different signalling pathways depending on the T cell subset (2). In Th1 cells, it primarily promotes STAT5 (signal transducer and activator of transcription 5) signalling, increases the expression of CD25, and redirects the signal to the PI3K/Akt/mTOR pathway. This shift causes intense anabolic metabolism, enabling the proliferation and maintenance of the effector function. In Tregs, Il-2 is essential for their development in the thymus but also for the proliferation and maintenance of Tregs at the periphery (81). In this subset, only the STAT5 transducing signal pathway is operant. PI3K/Akt/mTOR is inhibited by Phosphatase and Tensin homolog (PTEN), reducing mTOR kinase activity. Additionally, STAT5 is responsible for FoxP3 expression and upkeeping the suppressive capacity of Tregs (3, 82). The activation of the STAT5 pathway by Il-2 exerts a positive effect on the suppressive abilities of Tregs, but it also negatively regulates T follicular helper cells (Tfh) and Th17 cell differentiation, diminishing Il-17 production, which altogether prevents enhanced proinflammatory responses (83).

In contrast to Teffs, Tregs express a significant amount of high-affinity Il-2R, allowing them to respond to a low level of Il-2 stimulation. At the same time, a high amount of Il-2 showed to favour the development of Teffs (81). Hence, maintaining proper stimulation *via* the concentration of Il-2 is fundamental in establishing a balance between the progression of autoimmunity and health (83). These findings lead to the development of low doses of Il-2 treatment allowing for the prevention and proper control of autoimmune diseases (84).

## Autoimmune diseases and metabolism

Autoimmunity results from abnormal immune responses that occur against self-tissues and, in general, leads to excessive activation of inflammation and self-tissue damage. This stimulus brings a metabolic switch causing a Warburg-like up-regulation of aerobic glycolysis that adjust the balance between inflammatory and regulatory immune phenotypes (85). Again, also in this particular pathology, while classically activated Teffs require glycolysis for their survival and functioning, Tregs favour oxidative metabolism (86).

## Metabolome-influencing treatment of autoimmune conditions

Some drugs, commonly used to treat autoimmune diseases, influence the metabolism of immune cells. Glucocorticoids, like dexamethasone and corticosterone, inhibit glycolysis and lactate production hence decreasing T effector function in rat thymocytes (87). Another immunosuppressive agent, mycophenolic acid (MPA, an active form of mycophenolate mofetil-MMF), downregulates HIF-1α, which may positively influence Tregs development and suppressive capacity by inhibition of FoxP3 ubiquitination and degradation (68, 88). *In vitro* experiment on human CD4+ T cells showed that MPA inhibited proinflammatory Il-17, IFN-γ, and TNF-α production and reduced Akt/mTOR pathway signalling and STAT5 phosphorylation while Il-2 and CD25 were unaffected. Treatment with MPA also caused enhanced expression of FoxP3 (89).

A scientific effort is made to develop new treatment methods for autoimmune diseases. Some of them already utilize the modulation of metabolic pathways to change the immune response. As mentioned earlier, a lack of functional Tregs population can induce severe immune-mediated consequences. This information initiated a series of experiments using Tregs as potential therapeutic agents. Many clinical trials are conducted to assess the efficacy effects of Treg cell-based therapies for immune-related conditions, like Graft versus Host Disease (GvHD), graft rejections, Type 1 Diabetes (T1D), Multiple Sclerosis (MS), Systemic Lupus Erythematosus (SLE), Autoimmune Hepatitis, or Crohn Disease (15) (90).

For particular diseases (such as T1D) have entered clinical trials (91, 92). Ongoing standard protocols assume using polyclonal Tregs that are *ex vivo* expanded CD25+CD4+ cells (93, 94). This procedure aims to multiply the number of cells before the infusion (95). Safety of the treatment has been confirmed, and no serious adverse effects were reported in patients treated with polyclonal *ex vivo*-expanded autologous transfer of Tregs (90, 92, 96–98). The current focus is on improving Treg functionality, for example, producing antigen-specific Tregs by stimulation with monocytes (99) or co-administration of polyclonal Tregs with Il-2 (NCT02772679).

Influencing the metabolome of Tregs is another potential mechanism that can improve the stability and functionality of the cells used in treatment. Reports show that metabolism is the main factor behind maintaining the Treg/Th17 balance in SLE patients (100–102). These premises may be a basis for the future development of new methods to improve Treg function in immune-related conditions, probably even *in vivo*.

For example, the administration of dimethyl fumarate, a derivative of the Krebs cycle metabolite- fumarate, is widely used to treat autoimmune diseases such as psoriasis and multiple sclerosis (NCT01930708) (103). Procaccini et al. demonstrated that dimethyl fumarate (DMF) treatment increases the proliferative potential of Tregs in patients with relapsing-remitting multiple sclerosis (RRMS) and releases higher amounts of anti inflammatory Il-10. Additionally, the effect was exacerbated after anti-leptin monoclonal antibody (leptin neutralization) administration *in vitro*, which acts comparably to mTOR inhibition by rapamycin (104). These experiments compared Treg and T conventional cells purified from RRMS patients and healthy donors. Researchers used *in vitro* experiments to assess the proliferative and suppressive potential of mentioned subpopulations.

There are also data describing impairments in glucose metabolism, which are considered a key component in the pathogenesis of Rheumatoid Arthritis (RA). This is an autoimmune, chronic disease in which inflammation, mediated by T effector cells, affects the synovial tissue (105). Naive CD4+ T cells isolated from RA patients exhibit diminished glycolytic activity (106). The activity of lactate dehydrogenase is increased in fibroblast-like synoviocytes (FLS), causing lactate accumulation and a decrease in glucose concentration in joints, which also affects an immune response by impairing the suppressive function of Tregs (105). High lactate concentration in the inflamed tissues upregulates *Il17A* and *INFγ* mRNAs, while it does not change the expression of immunosuppressive Il-10 and TGFβ in CD4+ cells (59). The vital role in the upregulation of inflammation in high lactate conditions plays lactate transporter SLC5A12 as treatment with SLC5A12 antibody abolished the proinflammatory response (59). Additionally, lactate transported by the SLC5A12 transporter is implicated in the differentiation of Th17 T cell subsets. CD3/CD28 activated CD4+ cells taking up lactate showed an increased intracellular pool of citrate and acetyl-CoA, substrates for fatty acid synthesis (FAS). Results were confirmed by assessing the Acetyl-CoA-Carboxylase 1 (ACC 1; cytosolic isoform responsible for regulating fatty acid synthesis) and 5’AMP activated protein kinase (AMPK; enzyme regulating the process of fatty acid oxidation) activation levels, where ACC1 was increased, and AMPK activity was reduced in the presence of lactate (59).

These data support the concept that irregularities in glucose metabolism in the early stage of RA affect naive CD4+ T cells, which deviate from a regular differentiation pattern and commit to becoming pro-inflammatory effector cells (105, 107). In addition, some studies reported abnormal mitochondrial function in CD4+ T cells in patients with RA. This was described mainly as low ATP production and reduced reactive oxygen species (ROS) release (107).

Rapamycin (Rapa), also known as sirolimus, is a macrolide clinically used as an immunosuppressant in the treatment of patients after organ transplantation. Rapamycin interacts with mTOR (mTOR inhibition) and suppresses the growth and proliferation of T effector cells (108, 109). Interestingly, Rapa had the opposite effect on Tregs (1). Chen et al. showed that Rapa treatment not only promoted Tregs differentiation and proliferation but also increased FoxP3 expression and upregulated the mRNA level of PD-1 and immunosuppressive TGF-β in these cells (27, 110, 111). Additionally, data suggest that this macrolide may influence the metabolome of Tregs, as Rapa-treated cells showed a decreased concentration of glycolytic intermediates and increased ATP/ADP and ADP/AMP ratios in comparison to non-treated controls. This indicates the shift from the glycolytic-dependent to oxidative mitochondrial metabolism (OXPHOS), which improves the activity of Tregs (27).

A phase 2, single-centre, randomized, double-blind, placebo-controlled study was carried out, and results showed that Rapamycin treatment reduced insulin requirements in long-lasting Type 1 Diabetic patients (112).

Research on mice has shown that epidermal FABP (E-FABP) deficiency in CD4+ T cells suppresses Th17 through increased expression of peroxisome proliferator- activating receptor γ (PPARγ) while enhancing regulatory T cell development. This mechanism protected mice from autoimmune encephalomyelitis (EAE) in an experimental model of MS. This modulation may be an attractive way of metabolome-influenced treatment method for MS but also other autoimmune conditions (113).

In Systemic Lupus Erythematosus (SLE) patients, disease severity is associated with glutathione (GSH) depletion. Treatment with N-acetylcysteine (NAC), a GSH precursor, showed the inhibition of SLE progression by suppressing mTOR kinase and therefore, increase FoxP3 expression (114, 115). Research on NAC treatment in patients with SLE is currently in phase II clinical trials (116).

Several immunometabolism-related methods have been introduced to type 1 diabetes treatment (T1D). For example, the rapamycin and CD28 agonist combination aimed to inhibit T cell activation, migration, and pancreatic β-cell prevention (117). Another group tried to combine Rapamycin with Il-2, and islet autoantigen peptides, hence increasing the Treg number and protecting against T1D induction (118).

## Conclusion

The aim of metabolomics in medicine is to identify new markers, predict the onset, follow the progression of the diseases, and assess the efficacy of administered treatments (9). Metabolomics dictates possible modulations towards better regulation and tolerance. For example, switching cell metabolism from glucose-dependent to lipid-dependent pathways may improve the stability and functionality of Tregs while simultaneously suppressing the proinflammatory effects of Teffs. Moreover, the improvement in Treg function may also be obtained through influence on the main regulatory pathways, such as PI3K-Akt-mTOR, where suppression of these kinases results in the promotion of Treg proliferation, differentiation, and increased expression of FoxP3. Additionally, differences in cell metabolomes may serve as markers of the effectiveness of immunomodulatory treatment.

The above research raises hopes for treating autoimmune diseases by capturing pathological changes in cell metabolism and redirecting them into those favouring balanced regulatory properties.

## Author contributions

Conceptualization: MT Writing - original draft preparation: MT, AR Writing - review & editing: MZ, AJ-K, PT. All authors contributed to the article and approved the submitted version.

## Glossary

**Table:**

2-DG	2-deoxyglucose
ADO	adenosine
ADP	adenosine diphosphate
AMP	adenosine monophosphate
AR	adenosine receptor
AREG	amphiregulin
ATP	adenosine triphosphate
cAMP	cyclic AMP
CAR-T	chimeric antigen receptor T cell
CPT1	carnitine palmitoyltransferase-1
CTLA-4	cytotoxic T cell antigen 4
DGAT1	diacylglycerol O acyltransferase 1
DMF	dimethyl fumarate
FAO	fatty acid oxidation
FAS	fatty acid synthesis
GPR81	G protein-coupled receptor 81
GSH	glutathione
GLUT	glucose transporter
GvHD	Graft versus Host Disease
HIF-1a	hypoxia-inducible factor 1a
IFN-γ	Interferon-gamma
LDH	lactate dehydrogenase
MCT4	monocarboxylate transporter 4
MS	Multiple Sclerosis
MSC	mesenchymal steem cells
mTOR	mammalian target of rapamycin
NAC	N-acyetylocysteine
NF-κB	nuclear factor kappa-light-chain-enhancer of activated B cells
OXPHOS	oxidative phosphorylation
PEP	phosphoenolpyruvate
PEPCK	phosphoenolpyruvate carboxykinase
PGC1α/β	peroxisome proliferator-activated receptor- γ coactivator 1- α/β
PI3K-Akt-mTOR	Phosphoinositide 3-kinase- Protein B kinase- mammalian Target of Rapamycin
PIKK	phosphatidylinositol 3-kinase-related kinases
RA	Rheumatoid Arthritis
Rapa	Rapamycin
ROS	reactive oxygen species
RRMS	relapsing-remiting multiple sclerosis
SCFA	short chain fatty acids
SLE	Systemic Lupus Erythematosus
T1D	Type 1 Diabetes
TAG	tricacylglicerol
TCA	tricarboxylic acid cycle
TCR	T cell receptor
Teff	T effector cells
Tfh	T follicular helper cells
TGF-β	transforming growth factor β
TLR	Toll like receptor
TNF-α	tumor necrosis factor α
Tregs	T regulatory cells
